# Design and Mechanical Compatibility of Nylon Bionic Cancellous Bone Fabricated by Selective Laser Sintering

**DOI:** 10.3390/ma14081965

**Published:** 2021-04-14

**Authors:** Xuewen Chen, Tingting Lian, Bo Zhang, Yuqing Du, Kexue Du, Nan Xiang, Dong-Won Jung, Guangxin Wang, Akiyoshi Osaka

**Affiliations:** 1 School of Materials Science and Engineering, Henan University of Science and Technology, Luoyang 471023, China; lian_tingting@126.com (T.L.); zhangbo32103@126.com (B.Z.); DuyqStephanie@163.com (Y.D.); 180318020122@stu.haust.edu.cn (K.D.); xiangnan-87@163.com (N.X.); wgx58@126.com (G.W.); 2 Faculty of Mechanical, Jeju National University, Jeju Island 63243, Korea; 3 Institute of Engineering, Okayama University, Okayama 700-8530, Japan

**Keywords:** cancellous bone, honeycomb structure, selective laser sintering, equivalent modulus of elasticity, uniaxial compression, FEM

## Abstract

In order to avoid the stress shielding phenomenon in orthopedic bionic bone implantation, it is necessary to consider the design of mechanical compatible implants imitating the host bone. In this study, we developed a novel cancellous bone structure design method aimed at ensuring the mechanical compatibility between the bionic bone and human bone by means of computer-aided design (CAD) and finite element analysis technology (specifically, finite element modeling (FEM)). An orthogonal lattice model with volume porosity between 59% and 96% was developed by means of CAD. The effective equivalent elastic modulus of a honeycomb structure with square holes was studied by FEM simulation. With the purpose of verifying the validity of the cancellous bone structure design method, the honeycomb structure was fabricated by selective laser sintering (SLS) and the actual equivalent elastic modulus of the honeycomb structure was measured with a uniaxial compression test. The experimental results were compared with the FEM values and the predicted values. The results showed that the stiffness values of the designed structures were within the acceptable range of human cancellous bone of 50–500 MPa, which was similar to the stiffness values of human vertebrae L1 and L5. From the point of view of mechanical strength, the established cellular model can effectively match the elastic modulus of human vertebrae cancellous bone. The functional relationship between the volume porosity of the nylon square-pore honeycomb structure ranging from 59% to 96% and the effective elastic modulus was established. The effect of structural changes related to the manufacture of honeycomb structures on the equivalent elastic modulus of honeycomb structures was studied quantitatively by finite element modeling.

## 1. Introduction

At present, surgical implants made of medical stainless steel (SS316L), cobalt chromium (CoCr) alloy [1], titanium alloys (Ti-6Al-4V) [2] and magnesium (Mg10Zn4Y) alloy [3] have been used in the clinical operation of bone replacement. However, the implant of a completely compact, hard, artificial bone shows homogeneous behavior after surgery. When subjected to external load, the dense and hard bone implant bears more stress, which reduces the load transferred to the host bone, resulting in a change in the stress environment of the host bone, which is called a stress shielding phenomenon [4]. When stress shielding occurs in the bone tissue, the stress level on the bone is at a low level for a long time, which causes the bone tissue to gradually absorb, causing osteoporosis around the bone prosthesis and the loosening of the implant, leading to the patient’s pain [5]. Patients even need to undergo revision surgery again. Therefore, in order to avoid stress shielding during bone implantation, it is necessary to seek more flexible implants to achieve mechanical compatibility with the host bone.

For orthopedic implants, the mechanical compatibility between the implant and the host bone—that is, the match of the elastic modulus—is very important. Among the variety of biomechanical parameters of bone (strength, stiffness, creep, fatigue, permeability, etc.), the elastic modulus is an important parameter, essential for characterizing different kinds of skeletal pathologies and guiding the design of artificial implants [6]. Banhart [7] used traditional bone cement implants to provide a firm fixation for the implant through surface treatment of porous coatings, such as sintered beads, fiber mesh or thermal spraying. The results showed that this could provide sufficient stability. However, patients often complained of pain as a result of the mismatch of the equivalent elastic modulus between the rigid implant and the flexible bone tissue. Oshida’s research results showed that Ti-6Al-4V was used as the femoral stem in total hip arthroplasty. Due to the high elastic modulus (110 GPa) of the alloy, stress shielding showed in the results [8]. The study by Gepreel showed that when the low-stiffness bone was matched with the high-stiffness femoral stem, the implanted femoral stem would bear a considerable portion of the body load, resulting in the host bone being unable to generate the necessary stress to maintain its own stiffness, density and structural integrity [9]. Xu Yang’s research showed that when solid metal was implanted into the human body, it would not match the elastic modulus of the surrounding bones. When under loading, the prosthesis was too hard to bear more load, and the surrounding host bone did not have enough load to bear and was shielded by stress. Eventually, this would lead to the bone’s resorption and loosening and the offset of implantation and even fractures [10]. At present, the stress shielding effect that leads to osteoporosis is a difficult problem for biomaterials. In order to solve the stress shielding phenomenon, the effective solution is to adopt a porous structure. The design of the porous structure should consider imitating the structure of natural bone so that the mechanical properties of artificial bone are closer to the host bone, so as to promote the regeneration of bone tissue for bone repair. At present, there are researchers devoted to the study of the mechanical properties of porous titanium and titanium alloy implants. The apparent elastic modulus of NiTi with porosity of 16% measured by Greiner during uniaxial compression ranging from 15 to 25 GPa was similar to that of human bone [11]. Lin [12] used selective laser melting (SLM) to fabricate a topologically optimized Levine Ti-6Al-4V alloy lumbar interbody fusion apparatus. The average compression modulus of the experiment was 2.97 ± 0.90 GPa, having comparability with that of healthy cortical bone tissue. With an aim of reducing the stiffness of titanium implants, Harrysson designed cube structures with porosity ranging from 60% to 96.2% [13]. The elastic moduli of the titanium alloy tri-prism, four-prism and hexagonal prism cells designed by Wang were 41.12 GPa, 19.31 GPa and 9.07 GPa respectively, which is in line with the elastic modulus range of human cortical bone [2]. However, most of the porous titanium alloy structures designed in their study only match the elastic modulus of human cortical bone, while few researchers focus on matching the elastic modulus of cancellous bone.

At the same time, the porous structure of titanium alloy is not suitable to match the cancellous bone structure with high porosity and low elastic modulus [14]. When the elastic modulus of the porous titanium structure matches the high porosity, the thickness of the support rod is small, and the micro-cracks easily appear post-fabrication and after implantation. Titanium is sensitive to the cracks; therefore, it is easily broken if the implant is not sufficiently supported. The elastic modulus of the cellular structure is affected by structural characteristics and materials. The elastic modulus of nylon material is close to human cancellous bone, and its chemical structure and active group are similar to collagen protein; therefore, it has good biocompatibility with human tissue. Das [15] showed that Nylon-6 scaffolds prepared by selective laser sintering (SLS) provided excellent support for cell viability. Nylon can also show excellent mechanical properties due to strong hydrogen bonds between amide groups and Nylon macromolecules [16].

Boccaccio showed that the current design of porous scaffolds in bone tissue engineering is mainly a “trial and error method” involving the modification of the structural parameters of the design based on the test data in vivo or in vitro. This method requires expensive tests and a long time period [17]. However, Sun showed that computer-aided design (CAD) can help reduce the process of experimental testing and design of porous scaffold structures [18]. CAD includes the mechanical design of tissue engineering parts, which can design any structure required to meet the needs of the human body; this is significant for designing bone scaffold in tissue engineering. Adachi et al. found that, under many different types of design framework, a defined material (with elastic modulus and Poisson’s ratio given) could get a match to the strength or stiffness of natural tissues [19]. With the development of computing technology, we can not only use computers for structural design, but also use finite element analysis to solve mechanical analysis problems, such as structural strength, stiffness, elastoplasticity and so on. The practical application of finite element analysis to tissue engineering was first done in the design of bone scaffolds to optimize structural stiffness or geometric parameters. Jaecques [20] studied the internal stress and strain of the scaffold, as well as the mechanical response with the surrounding tissue, through finite element modeling (FEM). The results showed that a complete stress-strain analysis could be carried out. Cleynenbreugel showed that FEM could change multiple material or geometric parameters simultaneously, so as to obtain the most appropriate structure to replace the natural bone tissue of human body [21]. Therefore, the disadvantages of the traditional “trial and error method” in the design of porous scaffolds were avoided in this study. Advanced CAD and FEM can greatly reduce the number of experimental iterations and improve the performance of scaffolds.

To obtain verification of the accuracy of the finite element simulation and analysis method, the samples manufactured by SLS were tested by uniaxial compression to determine the effective stiffness characteristics of the porous structure. Harrysson studied the mechanical properties of a honeycomb model with a rhomboidal dodecahedron crystal structure manufactured by electron beam melting under uniaxial compression load, and compared the simulation results with the physical test results; the simulation results greatly exceeded the test results [13]. Parthasarathy [22] studied the effective stiffness of square-hole honeycomb structures with volume porosity between 45% and 75% under uniaxial compression load and the data showed that the simulation results exceeded the actual test stiffness again. The mechanical performance of honeycomb structures with higher porosity was more predictable. Smith studied the mechanical properties of a body-centric cubic model based on a stainless steel pore structure manufactured by SLM under uniaxial compression, and found that the simulated numerical results and the experimental ones had differences. By adjusting the structural parameters, there were no difference between the finite element simulation and experimental results [23]. Studies from the literature show that it is feasible to use the finite element simulation results in order to obtain a prediction for the effective mechanical properties of honeycomb structures [24]. Compared with physical testing, the finite element method can effectively reduce the test cost and shorten the test cycle.

To solve the stress shielding phenomenon existing in bone implantation, a stiffness match can be undertaken to effectively match the mechanical compatibility between orthopedic implant and host bone. In this paper, nylon was used as the material in a design of a honeycomb model with square holes and the effective elastic modulus of the honeycomb structure was studied by using the finite element method. The specimens prepared by SLS were tested by uniaxial compression to verify the effective compression performance of the honeycomb structure. The experimental results were analyzed and compared with the effective elastic modulus predicted by numerical simulation and analysis. The finite element model was used to simulate the structure change and study its effects on the effective stiffness.

## 2. The Design of the Honeycomb Structure with Square Holes

### 2.1. Design of Honeycomb Structure for Cancellous Bone

In the evolution of human bone, in order to ensure that the bone is lightweight, firm and can transport nutrients, many loose and porous cancellous bones have been formed inside and at the end of the bone. The cancellous bone presents a heterogeneous, porous and complex structure. The structure of biomimetic cancellous bone should ensure that the thickness, pore size and porosity of trabecular bone can meet that of natural cancellous bone. Therefore, the key parameters for the design of porous scaffolds are porosity, pore size, pore connectivity and bearing capacity. According to the previous studies, the pore size of cancellous bone is between 500–1000 μm, the porosity is between 50–90% [25], and the elastic modulus is between 50–500 MPa [26].

**Figure 1:**
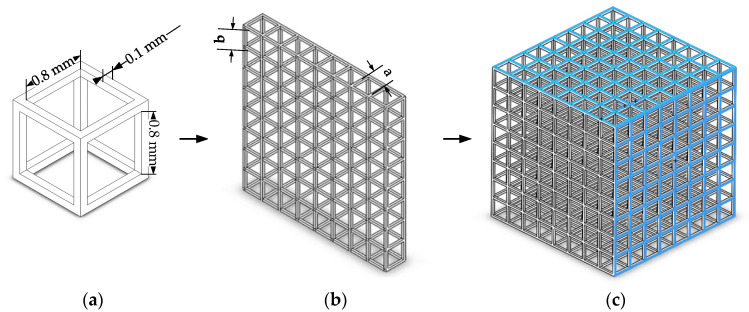
The 3D image of model 1 with the volume porosity of 70.45%. (**a**) Cube cell; (**b**) single-layer structure; (**c**) honeycomb structure.

In this study, a honeycomb structure model was established based on Diamant’s orthogonal lattice model for describing the cancellous bone of vertebrae by means of CAD. The pore structure of honeycomb structure model 1 is shown in Figure 1a. The internal aperture of honeycomb structure model 1 was a cube with a diameter of 0.8 mm, which was connected through a square channel with a diameter of 0.8 mm and a wall thickness of 0.1 mm. The structure sizes of the other three models are shown in Table 1. Using the linear pattern tool to repeat and transform the cell, single small cube units topologically form into a large cube honeycomb model with eight layers and eight columns, as shown in Figure 1c. As shown in Figure 1b, the central distance between points a and b of the adjacent scaffolds was controlled to remain unchangeable, and the wall thickness and pore diameter were changed to modify the porosity, so as to obtain models with different porosities of about 59% to 96%.

### 2.2. Finite Element Modeling of Honeycomb Structure

The finite element software was applied to predict the equivalent elastic moduli of four separate structures and the effect of the structure on their effective stiffness was studied.

#### 2.2.1. Prediction of Equivalent Elastic Modulus of PA66 Honeycomb Structure

**Figure 2:**
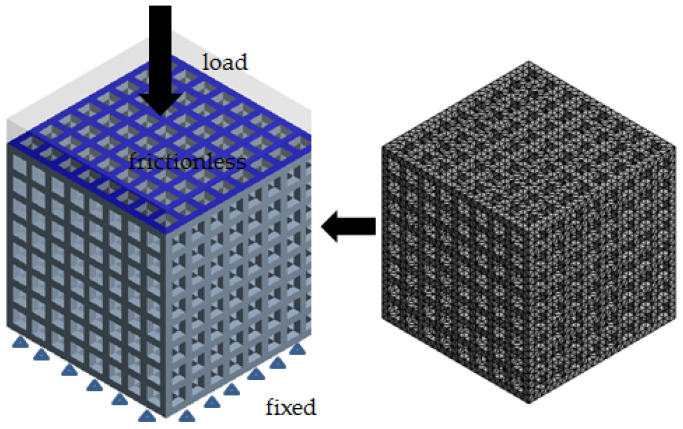
The finite element model of the honeycomb structure.

The compression performance of the scaffolds with different porosities was analyzed by finite element simulation to determine the optimal model with the appropriate porosity and safety factor. Usually, human vertebrae bear compression loads; therefore, a certain compression load can be applied to obtain the maximum deformation and stress of the model [27,28,29]. The loading model was created in the finite element software Ansys Workbench and the loading model was divided with automatic hexahedral meshes. The finite element model is shown in Figure 2. The load within the elastic limit of the material was applied on the cover slab, the top surface of the structure of which was gray and transparent. The bottom surface was subject to fixed constraints, such as that the triangle was a fixed load. The contact surface between the cover slab and the honeycomb model is shown in blue and frictionless constraints were applied. The simulation was carried out with the overall model. PA66 showed linear isotropic behavior with elastic modulus of 2.83 GPa and Poisson’s ratio of 0.4. Since the honeycomb structure was completely symmetrical in the three main axes of X, Y and Z, the elastic modulus of each direction had the following relation: E_X_ = E_Y_ = E_Z_. The equivalent elastic modulus *E_Q_* could thus be described by the following formula:

(1) $$E_{Q}=\sigma /\varepsilon =(F_{Z}/A)/(\Delta L_{Z}/L_{Z})$$

where $F_{Z}$ is the load applied to the end face in the *Z* direction and *A* is the cross-sectional area.

**Figure 3:**
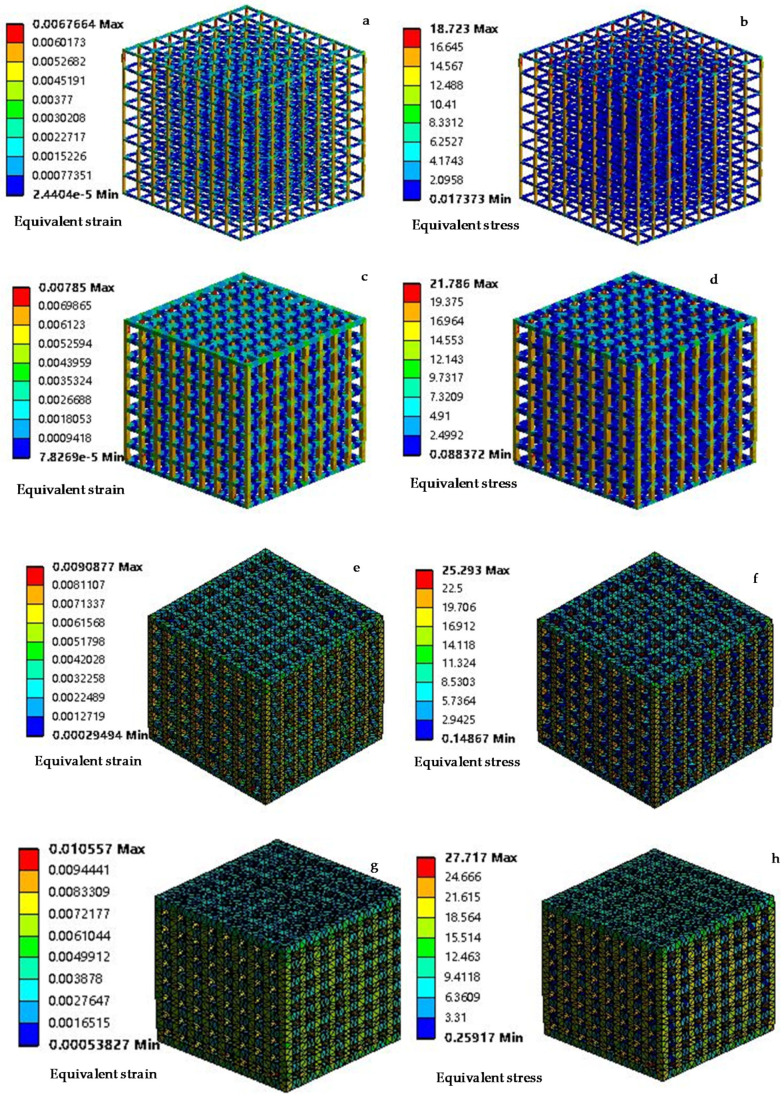
Calculation results of the deformation behavior (elastic deformation stage) of the four models. (**a**,**b**) Model 1; (**c**,**d**) model 2; (**e**,**f**) model 3; (**g**,**h**) model 4.

The distributions of stress (MPa) and strain of the four models are shown in Figure 3. With the increase of the pillar size, the porosity of the model decreases gradually, and the equivalent stress of the model entering the Mises yield state increases. According to the stress-strain distribution, the vertical pillar bears the main compression load. Large deformation and stress concentration appear at the junction of the trabecular beam.

#### 2.2.2. Modelling Structural Variation within a Honeycomb Structure

**Figure 4:**
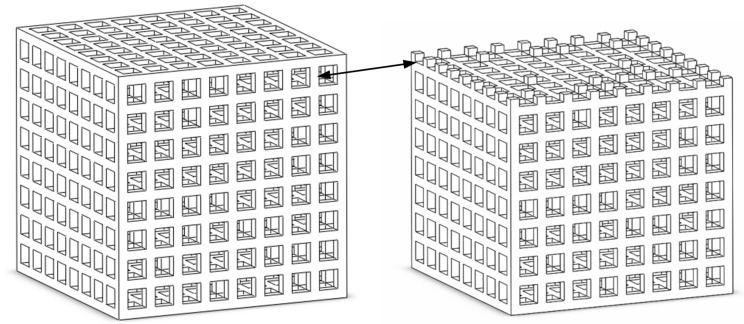
The honeycomb structure of the modified model 3.

**Figure 5:**
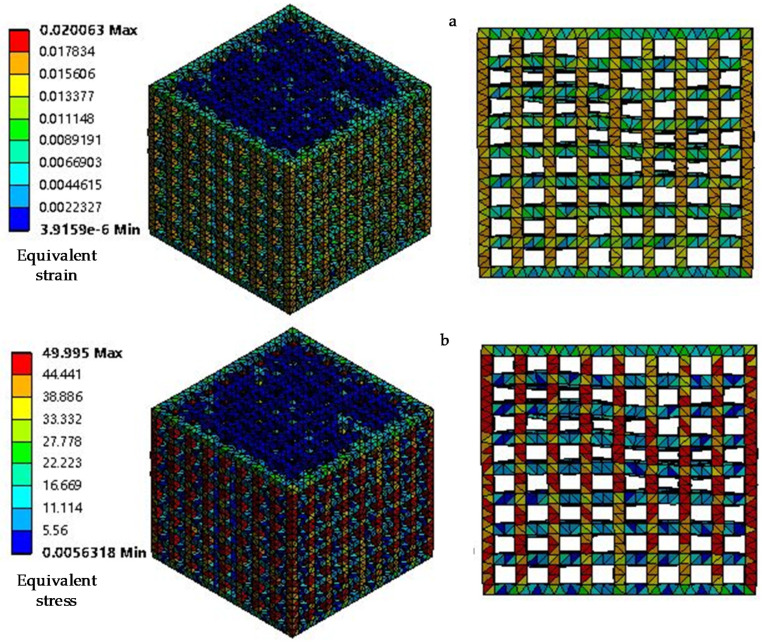
The distribution of equivalent stress and equivalent strain for model 3 with 256 pillars removed. (**a**) equivalent strain distribution; (**b**) equivalent stress distribution.

In this study, like in those of other researchers, we used a regular geometric structure and a perfectly smooth state to simulate the ideal state. However, the actual situation is that the cancellous bone of the human body is not in regular geometric shape, and osteoporosis occurs with changes in the body condition. Moreover, when the CAD model is manufactured by selective laser sintering technology, the structure changes and the surface finish is not smooth. Considering this situation, finite element modeling of internal structural changes in the model was carried out to study their effect on effective stiffness. To simulate the irregularity and heterogeneity of the porous structure, the stiffness of some parts was reduced by removing part of the strut directly. Figure 4 shows the modified finite element model for model 3 after 256 pillars were removed. In this case, the honeycomb model used the same loading conditions as before. In order to get a direct comparison of the effective stiffness, the adjusted model was compared with the ideal model. The distribution of stress (MPa) and strain for model 3 after 256 pillars were removed is shown in Figure 5. We can conclude from the figure that the loss of a pillar causes the local loss of support and the occurrence of large deformations.

### 2.3. Using the Classic Method of Gibson and Ashby to Predict the Effective Stiffness of the Cellular Structure of the Honeycomb Model

The compression mechanical characteristics of porous scaffolds are closely related to the porosity of the structure. In order to design a porous structure compatible with the stiffness of the host bone, the relationship between porosity and the equivalent modulus of elasticity should be studied quantitatively [30]. Gibson and Ashby (1997) studied the mechanical properties of porous structures with different porosities and developed the Gibson and Ashby model [31]. In order to compare physical test results and finite element simulation results, the classical Gibson and Ashby method was used to predict the porous structures’ effective stiffness [32]. The equation is as follows:

(2) $$E^{*}/E_{S}=C\left(\rho^{*}/\rho^{s}\right)$$

where $E^{*}$ is the hole structure’s elastic modulus and $E_{S}$ is the nylon solid material’s elastic modulus. *C* is the geometric proportionality constant, which is approximately 1 [33]. Equation (2) shows that the relative density determines the magnitude of the elastic modulus, and the relative density can be expressed with the porosity. The specific relationship is as follows:

(3) $$1-\theta =\left(\rho^{*}/\rho_{s}\right)$$

In Equation (3), $\rho^{*}$ is the density of the pore structure and $\rho_{s}$ is the density of the solid material.

According to the deformation of Equation (2), the equivalent elastic modulus can be expressed as follows:

(4) $$E_{e}=E_{s}{\left(1-\theta \right)}^{2}$$

Gibson and Ashby’s classical model has been used to predict the stiffness of open porous structures under vertical loads, which are similar to the structures studied in this paper. Therefore, the predicted finite element analysis results of Equation (4) and the subsequent test results were used for the comparative analysis.

## 3. Preparation for Uniaxial Compression Experiment

### 3.1. Selective Laser Sintering Technology

**Figure 6:**
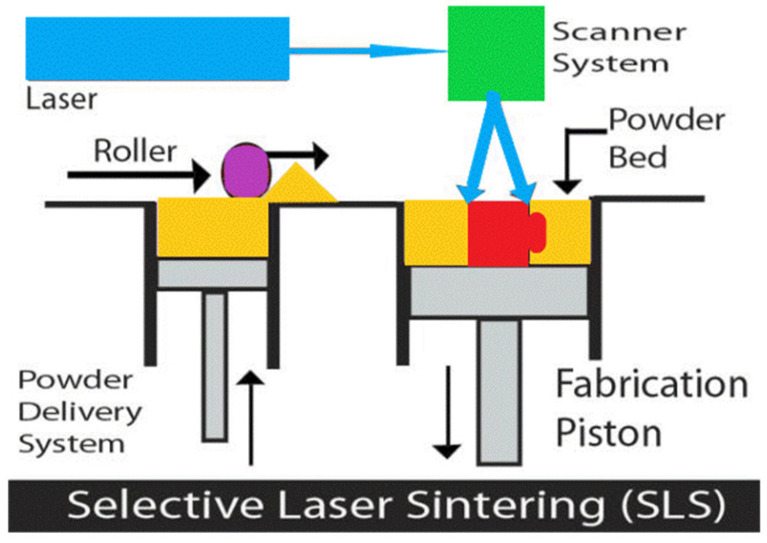
Principle of selective laser sintering technology.

**Figure 7:**
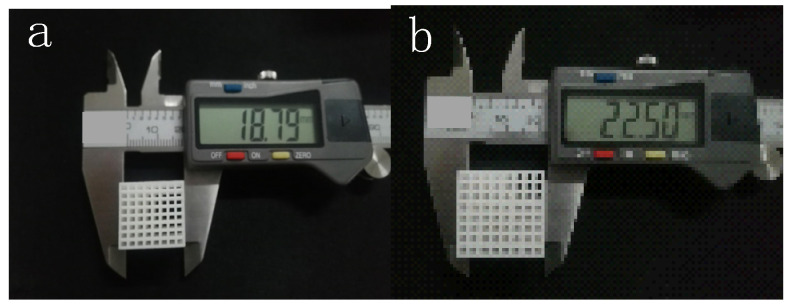
The 3D printed objects of model 3. (**a**) Model 3, m = 2.5; (**b**) model 3, m = 3.

Selective laser sintering technology is one of the best additive manufacturing technologies [34]. It uses high-power lasers to project onto the surface of the powder layer to achieve selective melting of powder materials. After each layer is melted, the printing platform drops by a thickness of one layer along with the production piston. The powder conveyor system then spreads the new powder onto the surface and pushes the powder flat and compacted through a roller. The laser beam is selectively sintered according to the cross-section information of layers under computer control, and then the next layer is sintered after the completion of one layer. The above process is then repeated until it is fused into a three-dimensional part to manufacture the entire part completely [35]. The working principle is shown in Figure 6. A porous scaffold printed by SLS can achieve minimum molding size, high quality, high strength, high precision and approximately 100% material utilization [36]. According to the research of Yan, the average size error of samples made by SLS is less than 1.37% in the x and y directions and 0.45% in the z direction [25]. Das [37] enlarged the micro-CT data of human proximal femoral cancellous bone by four times for selective laser sintering, and studied the strain value of the porous structure through a uniaxial compression test. In this paper, model 3 was printed at 2.5 times enlargement and triple enlargement. m is the enlargement scale factor of the scaled-up print relative to the original design model. The finished product is shown in Figure 7.

### 3.2. Stiffness Performance Testing of Honeycomb Structure

**Figure 8:**
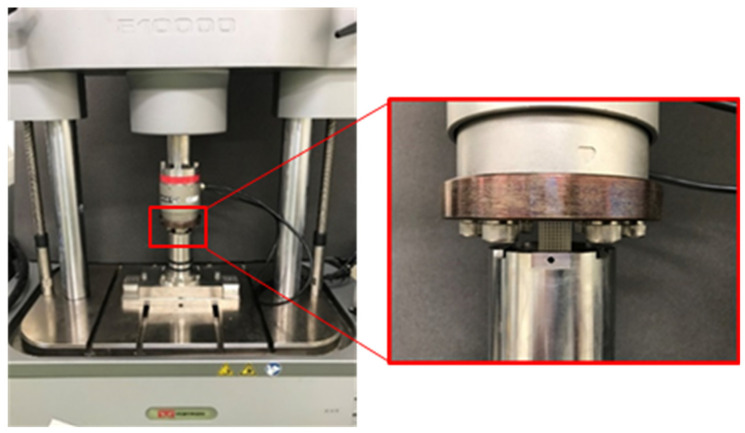
The Zwick Roell 1474 uniaxial compression test equipment.

The honeycomb structures manufactured by selective laser sintering were subjected to a uniaxial compression test on a Zwick Roell 1474 material testing machine with the maximum load capacity of 100 KN, as shown in Figure 8. In order to determine the compression performance of the honeycomb structure in the direction of its construction according to the JISK7181 standard [38], for which the test speed is the original length of the test samples multiplied by 0.02 mm/min, different samples were tested at 0.375 mm/min and 0.45 mm/min, respectively. The compression displacement was measured with the clamp on the extensometer. Based on the real-time force and displacement data obtained from the test equipment, the stress-strain relationship was calculated. Then, the equivalent elastic modulus was determined from the stress-strain curve.

## 4. Results and Discussion

### 4.1. Finite Element Simulation Results

**Figure 9:**
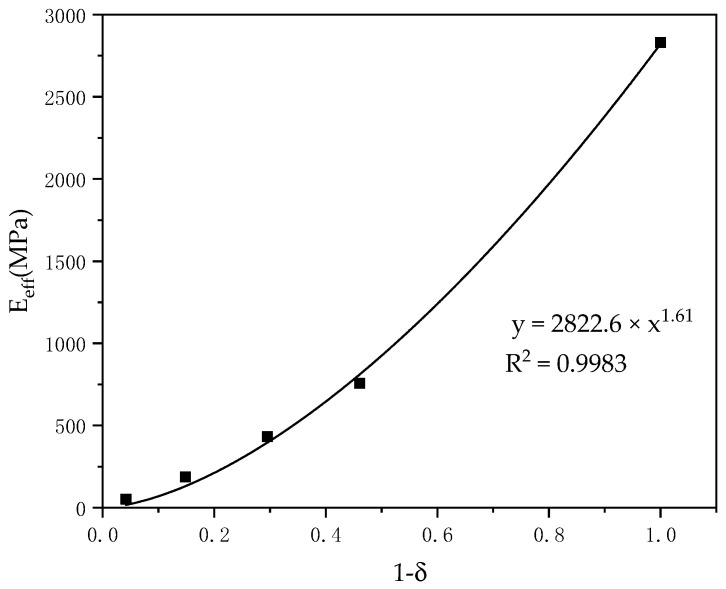
Relationship between equivalent elastic modulus and porosity.

Figure 9 shows that the stiffness value of the designed structure was 52.14–756.94 MPa, within the range of 50–500 MPa considered acceptable for human cancellous bone. The equivalent elastic modulus decreased with the increase of porosity. The relationship between the equivalent elastic modulus and the porosity is shown in Figure 9. The numerical results showed that there is a nonlinear relationship between the equivalent elastic modulus and the porosity of the honeycomb structure obtained by finite element simulation. Therefore, the nonlinear regression analysis method was used to study the relationship between the equivalent elastic modulus and porosity. According to the regression analysis, an equation was proposed to predict the equivalent elastic modulus of the honeycomb structure with square holes. The relationship is as follows:

(5) $$E_{\mathrm{eff}}/E_{S}=0.9974{\left(1-\delta \right)}^{1.61}$$

where $E_{S}$ is the equivalent elastic modulus of solid nylon 66 (PA66) and δ is the porosity. The judgement value (R^2^) coefficient of the derived expression was 0.9983, which can be seen in Figure 9. The porosity range applicable to this expression was greater than 50%. The research scope used by previous researchers obtained porosity values that were all greater than 50%. It can be observed from the data that, as the porosity decreases, the equivalent elastic modulus of the honeycomb model increases.

### 4.2. Effective Stiffness of Square-Pored PA66 Porous Structure Predicted by Gibson and Ashby Method

**Figure 10:**
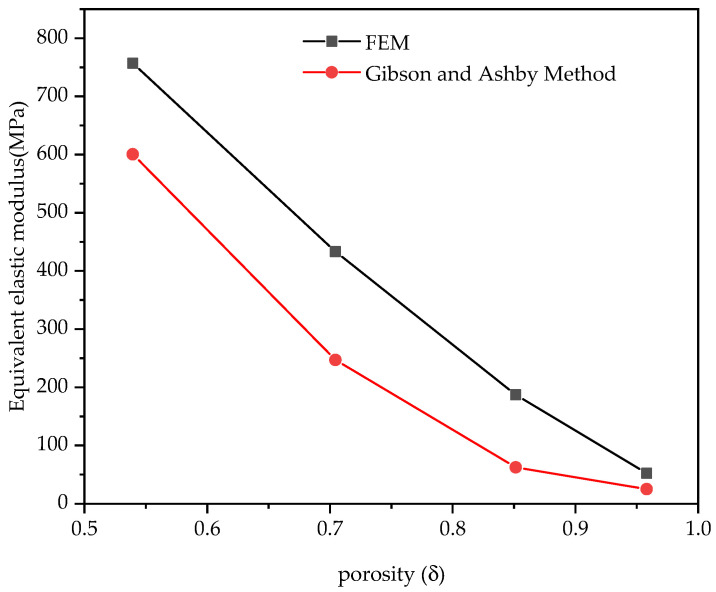
Comparison of the equivalent elastic moduli of the two methods for all models.

We predicted the equivalent elastic moduli of the four models by using Equation (4). As can be seen from Figure 10, the elastic modulus predicted by the Gibson and Ashby model method and the results of the finite element simulation had some similarities. Generally speaking, compared with the experimental value, the predicted value overestimates the stiffness of the structure, which is the conclusion drawn by most researchers [39]. As can be seen from the comparison results, the predicted value of the Gibson and Ashby method was very close to the finite element simulation value for the porous structure with high porosity. This may have been because the method was originally used to predict the effective stiffness of porous structures with porosities greater than 70% [31].

### 4.3. Uniaxial Compression Results

**Figure 11:**
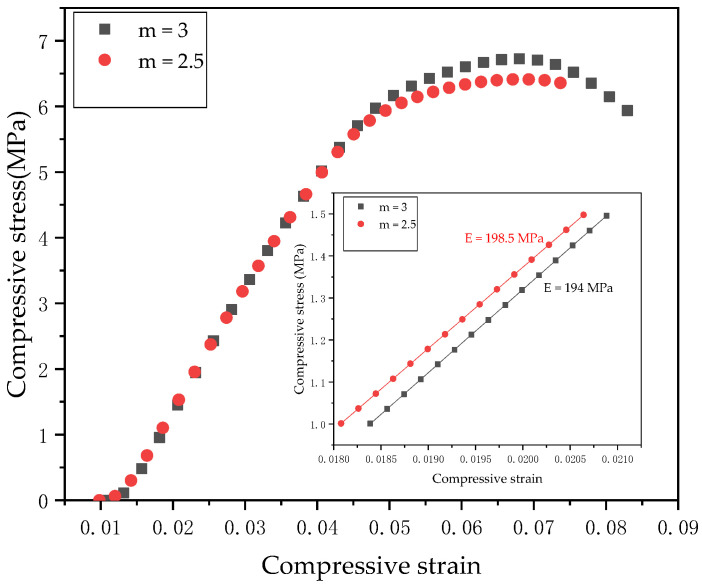
The stress and strain curve of the uniaxial compression test.

According to the real-time force and displacement data of the test equipment, the average compression characteristics and stress-strain curves of model 3 were calculated, as is shown in Figure 11. It can be seen from Figure 11 that linear elasticity appears in the state of low stress. With the increase of stress, the porous structure SLS sample rapidly reaches the yielding state from the original state. When their porosities have no difference, the equivalent elastic modulus and yield strength increase with the increase of strut size. However, the increase is not large and there are small differences. It can be seen from Figure 11 that, after the same scale has been expanded, the stiffness values under the same porosity are basically the same, and there is a small difference, so the size effect was ignored in this experiment. The mechanical properties of the honeycomb model depend on the mechanical properties of the material, and the detected modulus was lower than the true elastic modulus due to possible changes in the microstructure during the machining process and additional deformation due to slip or local slip at the load point. The reason for these phenomena may be that the smaller size of the support rod leads to structural changes and the overall structure becomes thinner, resulting in large deformation under a small load [22]. It has been reported that the stiffness values of cancellous bone of human vertebrae L1 and L5 are about 190 MPa [40]. The equivalent elastic modulus obtained from the test data was similar to the stiffness value of human vertebrae. In further studies, structures with porosities no less than 50% should be used to change the stiffness of cancellous bone used for human vertebrae. When implanting functionally graded porous structures, finite element methods should be used to study the stress distribution and improve the functional adaptability of the vertebrae.

**Figure 12:**
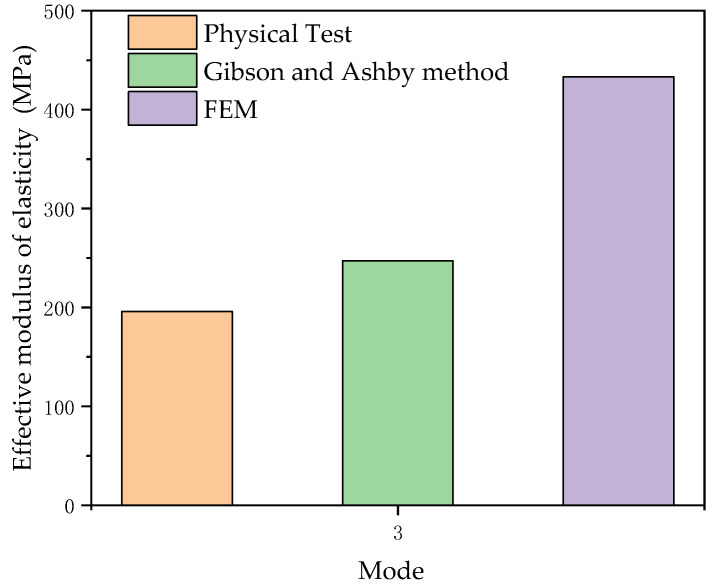
Comparison of equivalent elastic moduli of the three methods in model 3.

The comparison of the effective stiffness of model 3 obtained by different methods is shown in Figure 12. The finite element simulation and Gibson and Ashby method calculation were not completely consistent with the experimental values, as shown in Figure 10 and Figure 12. The data show that when the two groups of data were compared, the results of the numerical analysis were higher than the actual test values, and the average coefficient of data difference was 1.2. However, the connection of the calculated and the experimental results has some similarities with other studies. The trend of the Gibson and Ashby method analysis data is consistent with that of the finite element simulation data, and the change trend of the equivalent elastic modulus can be predicted. According to Hazlehurst’s study [41], within the acceptable range of 50–500 MPa, the average value of the error between a simulation and test results is smaller than the average difference coefficient of 3.3, which is obtained by dividing the error value between the FEM predicted value and the experiment by the compressed test value. Eshraghi examined the square pore structure of polycaprolactone manufactured by selective laser sintering [42]. By comparing physical analysis and numerical analysis, it was found that the two sets of data were in close agreement with the observed error of about 30%. This provides us with the idea that the mechanical properties of PA66 porous structures manufactured using SLS can be predicted. The differences between the experimental results and the simulation results shown in the current results can be attributed to factors related to the SLS manufacturing process of PA66, including cell body deformation and structural changes.

### 4.4. Results of Changes within the Honeycomb Structure

**Figure 13:**
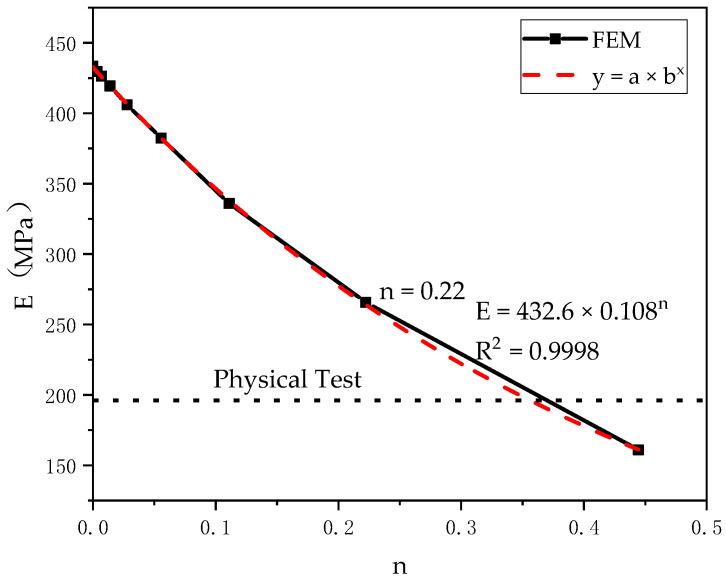
Effect of stiffness reduction coefficient n on equivalent elastic modulus.

In order to study the effect of structural changes on the effective stiffness of the model, the structural changes were applied to model 3. The method used in this work was to reduce the stiffness of selected elements based on the honeycomb structure. By this method, the randomly selected internal pillars were removed to reduce the stiffness and represent the structural changes and heterogeneity within the cell. n is the ratio of the pillars removed and we defined it as the stiffness reduction factor. The equivalent elastic modulus under different stiffness reduction coefficients is shown in Figure 13. The relationship between equivalent elastic modulus E and stiffness reduction factor n was obtained by fitting. This structural variation resulted in the calculated equivalent modulus of elasticity being lower than the ideal stiffness of the structure. The data showed that when the ratio of stiffness reduction was 22.22%, it was close to the measured value. In further research, the location and distribution of defects in cancellous bone could be determined by computed tomography, and the stiffness reduction factor could be adjusted to design a cellular honeycomb model for which the test value would have similarities with the equivalent elastic modulus of human cancellous bone. When defects in porous structures can be identified by using appropriate inspection methods, the percentage and distribution of elements used to reduce stiffness could be adjusted in further studies. It can be considered that the modeling technology has produced a new idea. It is proved that the structural variation and heterogeneity of honeycomb structures manufactured by SLS have great influence on the structural stiffness.

## 5. Conclusions

The goal of this study was to match the mechanical compatibility of an orthopedic implant effectively with the host bone to improve the osseointegration ability, and to address the stress shielding phenomenon existing in bone implantation. In order to verify the validity of the cancellous bone structure design method, the rigidity characteristics of the selective laser sintering square-hole honeycomb structure were designed and studied. The main results are as follows.

A honeycomb structure with volume porosity between 59% and 96% was designed. It could be observed through finite element simulation that the equivalent elastic modulus of the honeycomb model increased with the decrease of porosity. The finite element results showed that there was a nonlinear relationship between them. A formula for predicting the equivalent elastic moduli of honeycomb structures with square holes was presented.

The equivalent elastic modulus of a PA66 honeycomb structure manufactured by SLS was obtained with a uniaxial compression test and had similar stiffness characteristics to human vertebral cancellous bone, which has a value of 190 MPa.

A finite element model was proposed to prove the theory that the structural variation and heterogeneity of honeycomb structures manufactured by SLS can have an effect on the structural stiffness. At the same time, the difference between the finite element data and the experimental results was analyzed quantitatively. When the stiffness reduction factor n = 22.22%, the finite element data was consistent with the experimental results, which proves the effectiveness of the modeling method.

## Figures and Tables

**Table 1:** Dimensions and volume porosities of honeycomb structures.

Model	Strut Size (mm)	Pore Size (mm)	Porosity (%)
Model 1	0.1	0.8	95.81
Model 2	0.2	0.7	85.13
Model 3	0.3	0.6	70.45
Model 4	0.4	0.5	53.94

## Data Availability

Data available on request due to restrictions eg privacy or ethical. The data presented in this study are available on request from the corresponding author. The data are not publicly available due to these data are part of ongoing research.
